# Beyond FLAIR: PRISM (polarity-preserving real inversion solute mapping)—a new Frontier in real inversion recovery MRI for intracranial and sensory organ fluid assessment

**DOI:** 10.1007/s11604-026-02007-4

**Published:** 2026-05-30

**Authors:** Shinji Naganawa, Rintaro Ito, Yutaka Kato, Toshiaki Taoka

**Affiliations:** 1 Department of Radiology, Nagoya University Graduate School of Medicine, 65 Tsurumai-Cho, Shouwa-Ku, Nagoya, 466-8550 Japan; 2 Department of Innovative Biomedical Visualization (iBMV), Nagoya University Graduate School of Medicine, Nagoya, Japan

**Keywords:** Magnetic resonance imaging, 3D-real IR, PRISM, Gadolinium, FLAIR

## Abstract

Assessing subtle compositional changes in cranial and sensory organ fluids—including cerebrospinal fluid (CSF), ocular humors, and inner ear lymph fluid—is vital for neuroimaging. Heavily T2-weighted Fluid-attenuated Inversion Recovery (FLAIR) is sensitive to T1 changes induced by solutes, but conventional magnitude reconstruction (e.g., HYDROPS subtraction) suffers from artifacts like signal cancellation, paradoxical gadolinium-based contrast agent (GBCA) effects, and motion misregistration. This review summarizes the technical characteristics and diverse applications of PRISM (Polarity-preserving Real Inversion Solute Mapping), a robust 3D-real Inversion Recovery (IR) sequence developed to overcome these limitations. PRISM achieves high T1-sensitivity using phase-sensitive (real) reconstruction with an ultra-long repetition time (TR), allowing whole-brain coverage and simultaneous assessment of CSF, the eyeball, and the inner ear within a clinically feasible time. Critically, its polarity-preserving display depicts fluids lacking T1-shortening solutes with a negative signal (black), clearly differentiating them from bone and air. Clinically, PRISM’s utility spans both non-contrast and contrast-enhanced applications. Non-contrast PRISM is useful for assessing CSF protein concentration variations, meningeal lymphatic stasis, or compositional changes indicating inner ear pathologies. When combined with GBCA, time-course PRISM (pre-contrast up to 24 h delayed) uniquely visualizes solute dynamics, providing profound insights into blood-barrier integrity (e.g., blood-labyrinth barrier) and glymphatic waste clearance (e.g., CSF washout, perivascular space enhancement). Moreover, PRISM is a reliable, single-acquisition method for visualizing delayed contrast-enhanced endolymphatic hydrops, bypassing prior subtraction pitfalls. PRISM’s ability to detect subtle compositional markers with high resolution positions it as a highly promising MRI advancement. However, to facilitate its routine widespread clinical adoption, further standardization, broader validation, and multi-platform reproducibility are essential. Continued efforts to establish robust protocols will be necessary to realize its potential as a non-invasive tool for screening and monitoring glymphatic function.

## Imaging fluid solutes: from FLAIR to real inversion recovery

In the intracranial and head and neck regions, many fluid components other than blood exist, such as CSF in the ventricles and subarachnoid space, aqueous humor and vitreous humor in the eyeball, endolymph and perilymph in the inner ear, lymph fluid in meningeal lymphatic vessels, and interstitial fluid in the brain’s perivascular spaces [1–5].

FLAIR (Fluid attenuated Inversion Recovery) images are considered far more sensitive to changes in solute concentration within fluids than conventional T1-weighted or T2-weighted images [6, 7]. Heavily T2-weighted FLAIR, achieved by extending the Echo time (TE), is considered even more extremely sensitive to subtle changes in T1 value than conventional FLAIR used for routine brain parenchyma imaging [8]. Furthermore, extending the Repetition time (TR) in heavily T2-weighted FLAIR has been shown to further increase sensitivity to subtle T1-change in fluids [9, 10]. Since 2D-FLAIR is susceptible to inflow artifacts due to CSF movement, 3D-FLAIR is advantageous for evaluating CSF in the fast-moving basal cisterns [11, 12].

**Fig. 1:**
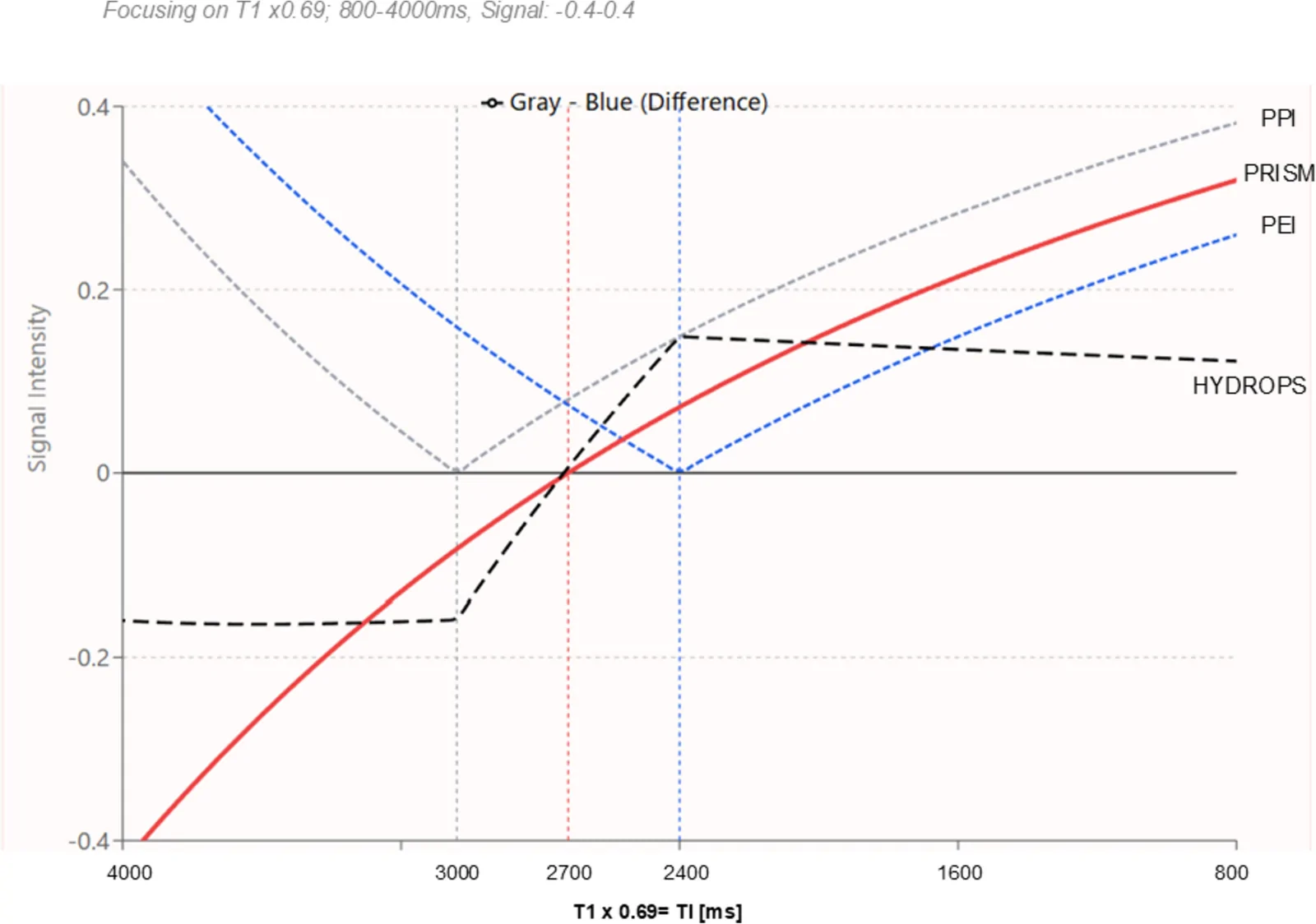
Conceptual diagram comparing the distribution of difference images from two magnitude reconstructions at different inversion times (TIs) and the signal changes in the intermediate TI with phase-sensitive reconstruction in the inversion recovery method. The gray dashed line represents the long TI Null (magnitude reconstruction). The blue dashed line represents the Short TI Null (magnitude reconstruction). The red solid line indicates the intermediate Null (phase sensitive reconstruction). The black dashed line shows the difference between the gray and blue dashed lines. This difference corresponds to the difference between the positive perilymph image (PPI, gray dashed line) and the positive endolymph image (PEI, blue dashed line) acquired for the evaluation of endolymphatic hydrops four hours after intravenous administration of a gadolinium-based contrast agent (GBCA). Under the recommended ultra-long TR condition of 15,000–16,000 ms, the inversion time is 2400 ms for the PEI, 3000 ms for the PPI, and 2700 ms for the PRISM (polarity-preserving real inversion solute mapping) sequence. For substances with T1 values falling within the region between TI of 2400 ms and 3000 ms, the response is highly sensitive (steep slope) and nearly linear, as indicated by the slope of the black dashed line. In contrast, the PRISM sequence (red solid line) shows less steep changes within this range but allows evaluation even outside the range

Difference images, such as those used in the HYDROPS (HYbriD of Reversed image of Positive endolymph signal and native image of positive perilymph Signal) sequence for inner ear endolymphatic hydrops evaluation [13] (derived by subtracting an image with a slightly shorter Inversion Time from a Magnitude reconstruction heavily T2-weighted FLAIR image), exhibit approximately twice the T1 sensitivity compared to the single image before differencing. Within the range bracketed by two null points, the signal change becomes nearly linear with the T1 change (Fig. 1). However, outside this two-null-point range, the linear relationship between the change in T1 and the change in signal does not hold [14]. The difference between two inversion recovery images with different inversion times (TIs) has been conventionally used as the HYDROPS method for evaluating endolymphatic hydrops after delayed contrast administration [13, 15].

**Fig. 2:**
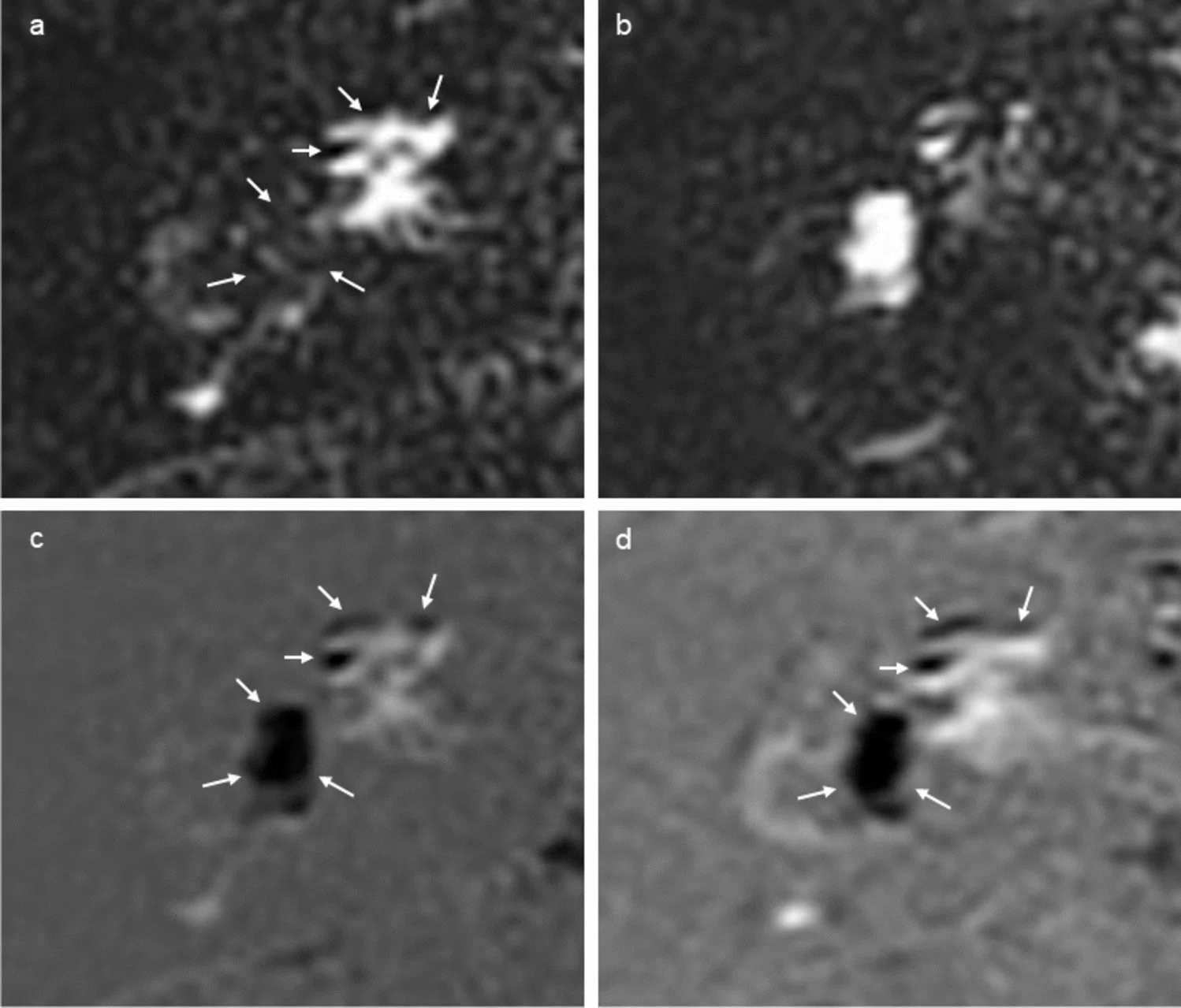
A woman with Meniere’s disease in her 30 s. Images are obtained 4 h after intravenous administration of single dose GBCA. **a** Heavily T2-weighted 3D-FLAIR image; the positive perilymph image (PPI, TR/TE/TI:16,000/544/2900). Perilymph shows bright signals by the distribution of GBCA. **b** The positive endolymph image (PEI, TR/TE/TI: 16,000/544/2500). Endolymph without the distribution of GBCA shows bright signals. **c** HYDROPS (HYbriD of reversed image of positive endolymph signal and native image of positive perilymph signal) is generated by the subtraction of PEI from PPI.Separation between bright perilymph, black endolymph, and gray bone is possible on a single image. Significant endolymphatic hydrops is observed in the cochlea and the vestibule (arrows). **d** The 3D-real IR, PRISM (TR/TE/TI: 16,000/538/2700). Endolymphatic hydrops in the cochlea and the vestibule (arrows) are visualized almost similarly as HYDROPS image. Note that the boundary between the endolymph (short arrows) and the adjacent bone and air is indistinct on the 3D-FLAIR image (**a**), whereas it is clearly delineated on the HYDROPS (**c**) and PRISM (**d**) images. The images are reprinted from the reference under the Creative Commons Attribution-NonCommercial-NoDerivatives International License [16]. GBCA, gadolinium-based contrast agent; PRISM, Polarity-preserving Real Inversion Solute Mapping

**Fig. 3:**
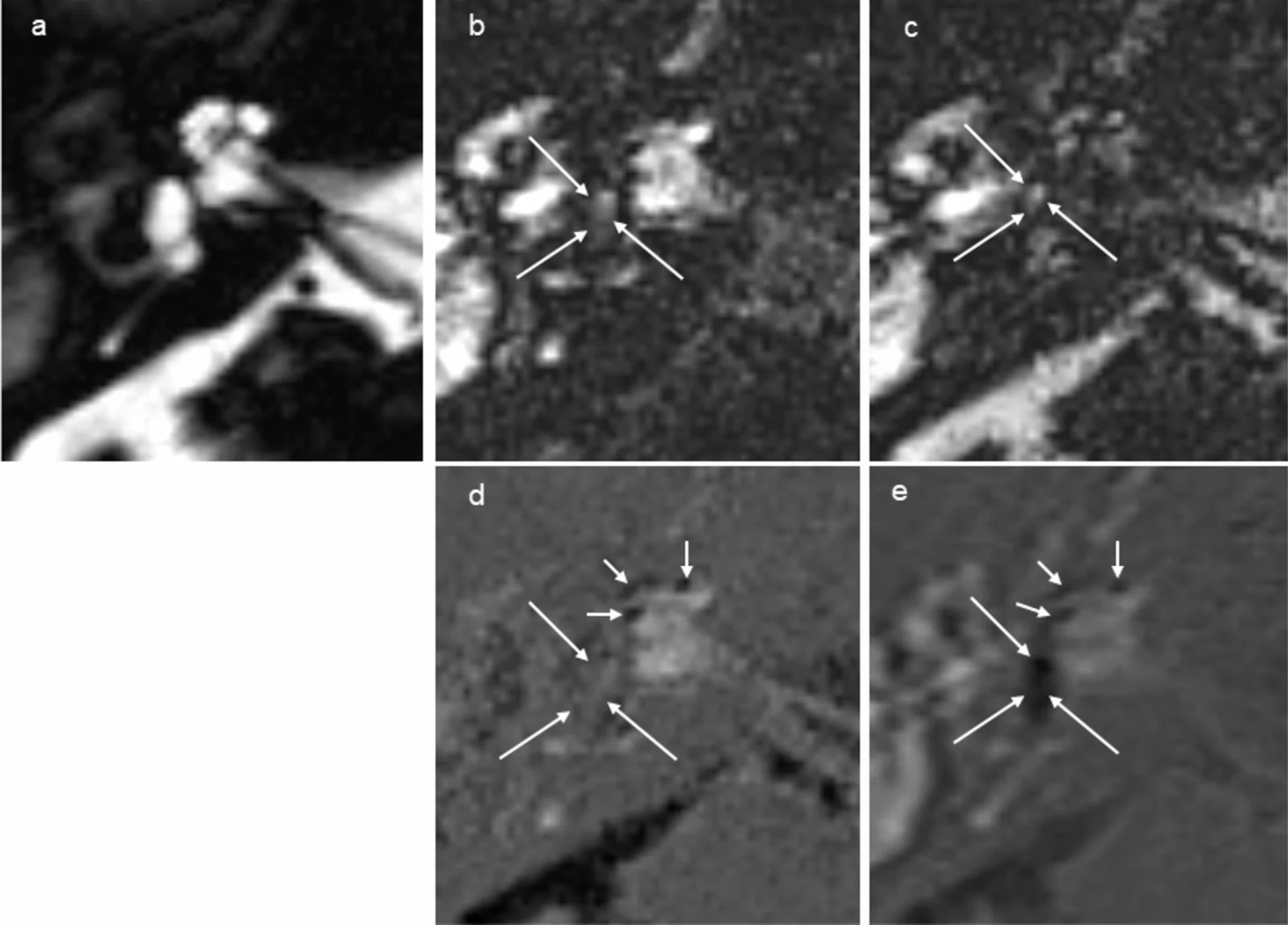
A man in his 70 s with Meniere’s disease with otitis media. Images were obtained 4 h after intravenous administration of GBCA. **a** MR cistertnography shows the whole fluid space of the right inner ear. **b** The positive perilymph image (PPI, TR/TE/TI:16,000/544/2900). Slight high signal area (long arrows) is seen in the center of the vestibule. **c** The positive endolymph image (PEI, TR/TE/TI: 16,000/544/2500). Slight high signal area (long arrows) is seen in the center of the vestibule. **d** HYDROPS (HYbriD of reversed image of positive endolymph signal and native image of positive perilymph Signal) image is generated by the subtraction of PEI from PPI. Endolymphatic hydrops in the vestibule is not visualized as black area. A small amount of GBCA is presumed to have distributed into the vestibular endolymphatic space—which typically has minimal GBCA distribution—likely due to inflammation. This distribution causes the signal in the vestibular endolymphatic space to increase on the PPI and decrease more than usual on the PEI. Consequently, the signal in the vestibule becomes nearly zero after the subtraction (HYDROPS), making the endolymphatic space difficult to recognize on the image. Endolymphatic hydrops in the cochela is clearly visualized (arrows). **e** 3D-real IR, PRISM (TR/TE/TI: 16,000/538/2700). Endolymphatic hydrops in cochlea (arrows) are visualized almost similarly as HYDROPS image. Endolymphatic hydrops in the vestibule is visualized on this image (long arrows). The images are reprinted from the reference under the Creative Commons Attribution-NonCommercial-NoDerivatives International License [16]. GBCA, gadolinium-based contrast agent; PRISM, Polarity-preserving Real Inversion Solute Mapping

However, Magnitude reconstruction of images acquired with the inversion recovery (IR) method can lead to a paradoxical signal decrease depending on the distribution of gadolinium-based contrast agent (GBCA), and an artifact known as the signal cancelation band may appear at the interface between two compartments with positive and negative magnetizasion. Furthermore, if a small amount of GBCA distributes into the endolymph—which typically has minimal GBCA distribution—due to inflammation or other effects, the endolymph signal increases in the positive perilymph image of Magnitude reconstruction, and the endolymph signal decreases more than usual in the positive endolymph image. This causes the difference, which is normally expected to be a negative signal, to become zero, a phenomenon that makes the endolymphatic space difficult to recognize [16] (Fig. 2, 3). Naturally, the subtraction of two images always carries the risk of misregistration due to patient motion between the two acquisitions. In the IR method using Phase sensitive reconstruction (real reconstruction), T1 differences can be emphasized even in a single acquisition by utilizing phase information [17].

To address the above-mentioned issues, long TR heavily T2-weighted 3D-real IR has been utilized over the past decade for the detection of subtle compositional changes in fluid components. Its utility in detecting subtle T1 value changes in fluid components has already been demonstrated in various fields [18–24]. In this paper, we namse this sequence PRISM (Polarity-preserving Real Inversion Solute Mapping) and review its applications and characteristics in neuroimaging.

## Technical principles of PRISM and the difference from FLAIR

In the IR method, phase-sensitive reconstruction (also referred to as real reconstruction) utilizes phase information to emphasize T1 differences within a single acquisition. A 3D pulse sequence utilizing this technique is termed 3D-real IR. In this paper, we refer to our specific implementation—an ultra-long TR, heavily T2-weighted 3D-real IR sequence optimized for detecting subtle fluid compositional changes—as PRISM.

To implement PRISM using Ultra long TR heavily T2-weighted 3D-FLAIR, one only needs to slightly shorten the TI and then use phase sensitive (real) reconstruction. In other words, since fluid compositional changes that can be evaluated with Ultra long TR heavily T2-weighted 3D-FLAIR are, in most cases, also possible with PRISM, this paper will also summarize the current applications that have been achieved using Ultra long TR heavily T2-weighted 3D-FLAIR.

**Fig. 4:**
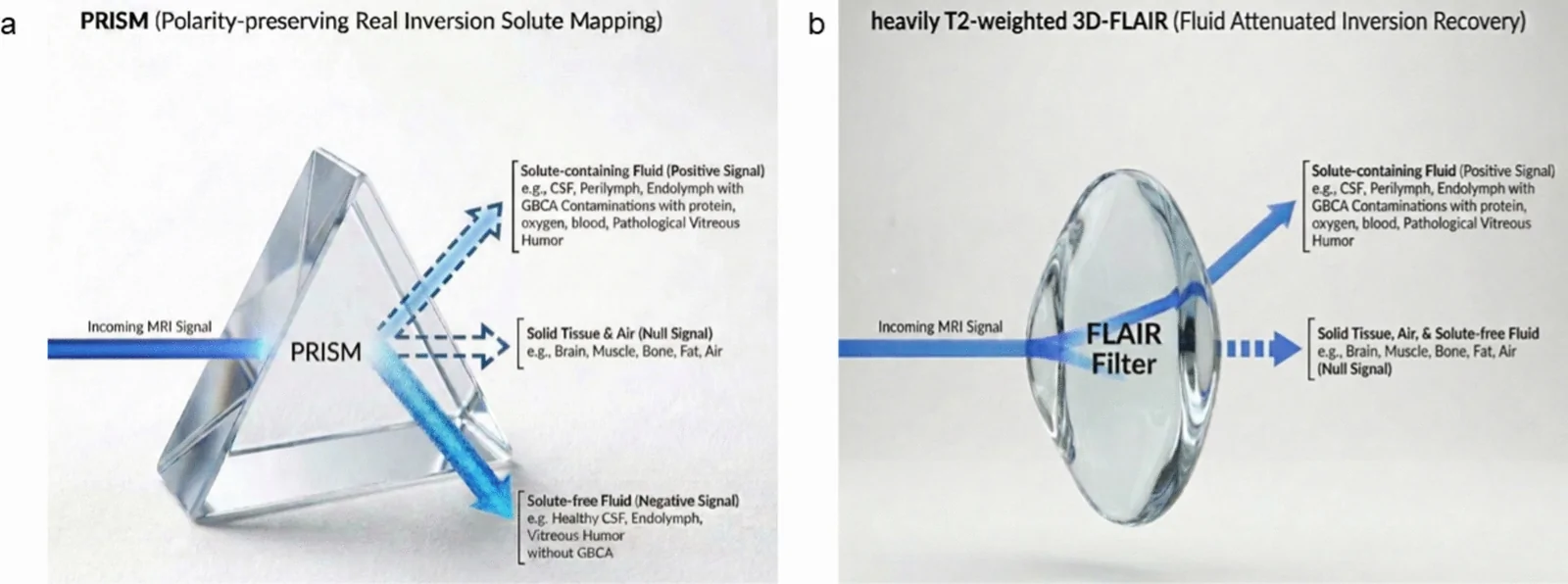
Conceptual diagram of the PRISM and FLAIR sequence. In PRISM (**a**), fluids containing solutes that alter T1 relaxation and those without such solutes are assigned to positive and negative signal polarities, respectively. Soft tissues such as normal brain parenchyma, which do not exhibit extremely long T2 values, as well as air, flow voids, and fat, are displayed in gray. As a result, PRISM is essentially a three-color rendering scheme. In contrast, heavily T2-weighted FLAIR (**b**) depicts water without solutes, soft tissues, bone, and air with similarly low signal intensity near zero (black on magnitude reconstruction), whereas water containing solutes appears hyperintense, resulting in a two-color representation. PRISM, Polarity-preserving Real Inversion Solute Mapping; FLAIR, Fluid-Attenuated Inversion Recovery

An important difference between heavily T2-weighted 3D-FLAIR and PRISM is that in FLAIR, fluids without T1-shortening solutes are displayed as black with a near-zero signal on images, making them difficult to distinguish from bone and air. However, in PRISM, fluids lacking T1-shortening solutes result in a negative signal, which allows for easy differentiation from bone and air (Fig. 2, 4). The features of PRISM are summarized in Table 1. A schematic diagram is shown in Fig. 4.

**Table 1:** Key features and advantages of PRISM (polarity-preserving real inversion solute mapping)

Feature	Description
Highly sensitive detection	It can sensitively detect low concentrations of GBCA (gadolinium-based contrast agent) or proteins in fluids, more sharply than T1-weighted images or conventional FLAIR images with moderate TR and TE
Reconstruction method	Since Phase sensitive reconstruction (real reconstruction) is used, paradoxical signal decrease caused by GBCA, as seen in Magnitude reconstruction, does not occur
Signal characteristics (fluid/tissue)	By slightly shortening the TI by approximately 200 ~ 400 ms from the CSF null point, CSF and endolymph are displayed as negative signals (black), distinguishing them from bone and air, which have zero signal. As the concentration of solutes that shorten the T1 value in the fluid (such as GBCA) increases, the signal changes from black to white
Signal characteristics (tissue/other)	Being a heavily T2-weighted image with a long echo time, brain parenchyma and bone exhibit almost no signal, showing near-zero signal values. In contrast, structures with long T2 times, such as the nasal mucosa, parts of the choroid plexus, and the pyramidal tract, are displayed as whitish positive signal values
SAR (specific absorption rate)	Because the TR (repetition Time) is very long (typically 15 s or more), even with factors that increase SAR, such as Adiabatic pulses for uniform inversion, fat saturation pulses, and long echo trains, the SAR per unit time is kept considerably lower than the limit, posing no impediment to imaging
Flow artifact	Since a large slab covering the entire cranium is inverted and excited at once, CSF flow artifacts (artifacts caused by flow) are unlikely to occur
Misregistration artifact	Unlike HYDROPS images used for evaluating endolymphatic hydrops of the inner ear, which involve subtracting two images acquired at different timings, misregistration artifacts (artifacts due to positional shift) caused by patient motion between the two scans do not occur

The purpose of this review is to introduce and summarize the characteristics of the PRISM sequence, its differences from FLAIR, and its utility in clinical practice and research, focusing particularly on the brain and sensory organs, based primarily on information from papers published to date.

## Pulse sequence for PRISM acquisition

### Imaging technique requirements

In conventional 3D-turbo spin echo (TSE) with an echo-train length of only a few tens, the Repetition Time (TR) cannot be sufficiently extended to cover a wide imaging range while keeping the acquisition time practical [25]. Even if the slab thickness is reduced to narrow the imaging range, the clinically usable TR is limited to at most about 6-9 s [25]. To increase the sensitivity to subtle compositional changes in fluids, an ultra-long TR—typically 15 s or more, and at least exceeding 10 s—is necessary to ensure sufficient longitudinal relaxation of the fluid. [9]. To cover the entire brain under this condition and maintain a clinically feasible acquisition time, a SPACE-like pulse sequence capable of using an extremely long echo-train length is required, along with the combination of acceleration techniques such as parallel imaging and compressed sensing [16]. By covering the whole brain, it is possible to simultaneously evaluate the CSF spaces and the eyeball, which are thought to reflect glymphatic system function, in addition to the inner ear [18–20, 26–28]. Noise reduction through AI-assisted reconstruction and spatial resolution improvement via super-resolution techniques would also be helpful. Non-uniform inversion within the Field-of-View (FOV) due to B1 inhomogeneity is a crucial challenge for the highly sensitive 3D-real IR sequence used in PRISM sequence. Efforts have been made to improve the method of applying the IR pulse to achieve uniform inversion within the FOV without significantly increasing the Specific Absorption Rate (SAR), as a countermeasure against B1 inhomogeneity [29, 30]. Fortunately, in PRISM, the SAR is typically not an issue because the TR is consistently maintained at the recommended ultra-long setting of 15 s or more. Most reports to date have been at 3 T. The typical scan parameters for the PRISM sequence at 3 T are shown in Table 2. The side-by-side comparison of conventional FLAIR, HYDROPS-type subtraction methods, and PRISM is shown in Table 3.

**Table 2:** A typical pulse sequence parameters for PRISM (polarity-preserving real inversion solute mapping) at 3 T

Sequence name	Type	Repetition time (ms)	Echo time (ms)	Inversion time (ms)	Flip angle (degree)	Section thickness/gap (mm)	Pixel size (mm)	Number of slices	Echo train length	Field of view (mm)	Matrix size	Number of excitations	Scan time (min)
3D-real IR	SPACE with inversion pulse	15,130	549	2700	90/constant145	1/0	0.5 × 0.5	256	256	165 × 196	324 × 384	1	10

GRAPPA (GeneRalized autocalibrating partial parallel acquisition) × 3 for real IR

SPACE: sampling perfection with application-optimized contrasts using different flip angle evolutions

This sequence utilizes a frequency selective fat suppression pre-pulse, non-slab selective excitation pulse, and non-slab selective inverison pulse

3D slab is set in an axial orientation

3D-real IR: 3D inversion recovery with phase sensitive reconstruction (real reconstruction)

**Table 3:** Comparison of imaging techniques for fluid assessment

Feature	Heavily T2-weighted 3D-FLAIR	HYDROPS (subtraction method)	PRISM (polarity-preserving real inversion solute mapping)
Acquisition strategy	Single TI setting with long TR	Two separate acquisitions with different TIs (e.g., PEI and PPI)	Single acquisition with intermediate TI and ultra-long TR (> 15 s)
Reconstruction method	Magnitude reconstruction	Subtraction of two magnitude-reconstructed images	Phase-sensitive (real) reconstruction
Main advantages	Simple clinical implementation; high sensitivity to T1-shortening effects	High T1-sensitivity; nearly linear signal change between two null points	High sensitivity in a single scan; no motion misregistration; clear differentiation of fluid from bone/air via signal polarity
Common pitfalls	Fluids without solutes appear black (zero signal), making them indistinguishable from bone or air	Risk of motion misregistration between scans; paradoxical signal decrease; signal cancellation bands at interfaces	Highly sensitive to B1 inhomogeneity; incomplete inversion near bone may lead to signal polarity errors

HYDROPS: HYbriD of reversed image of positive endolymph signal and native image of positive perilymph signal

PEI: Positive Endolymph Image, PPI: Positive Perilymph Image

### Technical challenges and optimization

The T2-prep technique [31–33] is useful for shortening the TR without significantly affecting SNR or contrast, but artifacts resulting from imperfect inversion are strongly expressed near bone. This can be particularly problematic for diagnosis in the inner ear and near the skull base, regions surrounded by bone [34–36]. Although technical developments are advancing to reduce the sensitivity of T2-prep techniques to B1 inhomogeneity, spin inversion remains incomplete in the vicinity of bone [37, 38].

In phase-sensitive (real) reconstruction, signal cancelation bands—which can occur in Magnitude reconstruction at the interface between two adjacent fluid compartments with nearly equal magnetization magnitudes of opposite signs—do not appear. For instance, because the cochlear endolymphatic space is small, the presence of a signal cancelation band around it can lead to an overestimation, making the endolymphatic space appear larger than its true size. However, some studies have intentionally leveraged the use of magnitude reconstruction to utilize the enhanced detection sensitivity of the cochlear endolymphatic space resulting from the appearance of the signal cancelation band [39, 40].

PRISM requires an ultra-long TR (> 15 s) to maximize T1 sensitivity, which inherently increases scan time. While parallel imaging and compressed sensing are employed to keep acquisition within 10–15 min, this remains longer than standard clinical sequences. Future integration of AI-assisted reconstruction and super-resolution techniques is crucial to further reduce scan time without sacrificing the signal-to-noise ratio. Additionally, B1 inhomogeneity remains a significant hurdle, particularly at the skull base and inner ear. Although phase-sensitive reconstruction eliminates signal cancellation bands, local field variations can still shift the “null point,” potentially leading to misinterpretation of fluid polarity if not carefully calibrated.

## Utility of non-contrast imaging

In PRISM images, CSF, vitreous humor, aqueous humor, and inner ear lymph fluid are typically depicted as black, i.e., negative signals. Air shows a signal of zero, and brain parenchyma is depicted as gray, with a signal close to zero. When fat saturation is used, fat is also close to zero, but signal remnants may be seen at the edge of the Field of View (FOV) due to incomplete suppression. The choroid plexus, perivascular spaces (PVH, white matter PVS) are depicted as white. A layer of high signal is also observed on the surface of the gray matter, which may reflect the signal of the subpial space.

### Non-contrast assessment of glymphatic/lymphatic pathways

**Fig. 5:**
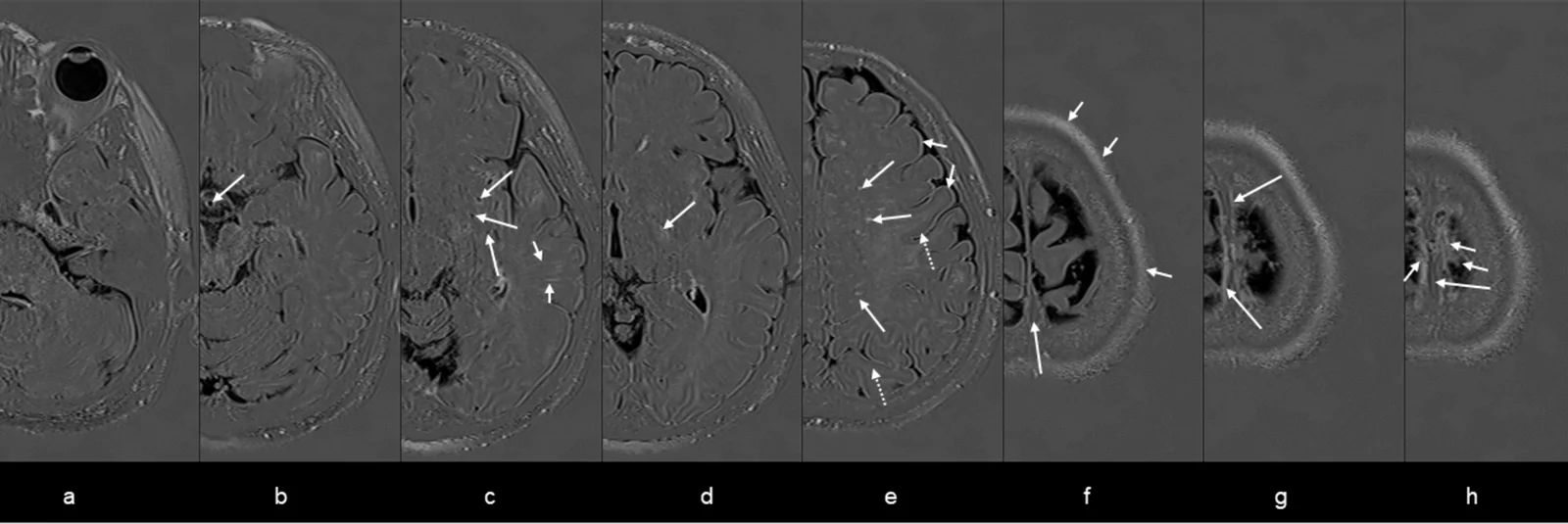
A woman in her 40 s who underwent MR imaging for dizziness, with no evidence of endolymphatic hydrops. Non‑contrast PRISM brain images are shown from the skull base to the vertex. **a** At the level of the internal auditory canal, the anterior chamber and vitreous body of the eyeball are depicted as hypointense and appear black. The inner ear structures and cerebrospinal fluid (CSF) in the cerebellopontine angle are also hypointense and appear dark. **b** At the level of the interpeduncular cistern, CSF appears black, and the cerebral aqueduct is also depicted as hypointense. The infundibular recess shows a dark signal within its lumen (arrow). The brain parenchyma, including both gray matter and white matter, is depicted as nearly uniform gray. **c** At the level of the basal ganglia, perivascular spaces within the basal ganglia are depicted as hypointense and appear black (arrows). In contrast, perivascular spaces in the cerebral white matter of the temporal lobe appear hyperintense (short arrows). The choroid plexus in the trigone of the lateral ventricle is depicted as hyperintense. **d** At the level of the posterior limb of the internal capsule, the corticospinal tract is depicted as faintly hyperintense (arrow). **e** At the level of the centrum semiovale, white matter lesions are depicted as hyperintense (arrows). The superficial layer of the cerebral cortex appears as if covered by a faint, thin hyperintense membrane (short arrows). In regions where the white matter signal is relatively high, the gray matter appears slightly hypointense in comparison (dotted arrows). **f** At the level of the inferior margin of the superior sagittal sinus, the venous lumen appears gray (arrow). Residual incomplete fat suppression is observed in the subcutaneous fat at the vertex (short arrows). **g** On the slice immediately superior to (**f**), structures presumed to represent meningeal lymphatic vessels along the superior sagittal sinus are depicted as hyperintense (arrows). **h** On the most superior slice, the parasagittal dura is depicted as faintly hyperintense (short arrows). A faint linear hyperintense structure within the lumen center of the superior sagittal sinus is presumed to represent the chordae Willisii (arrow). PRISM, Polarity-preserving Real Inversion Solute Mapping

**Fig. 6:**
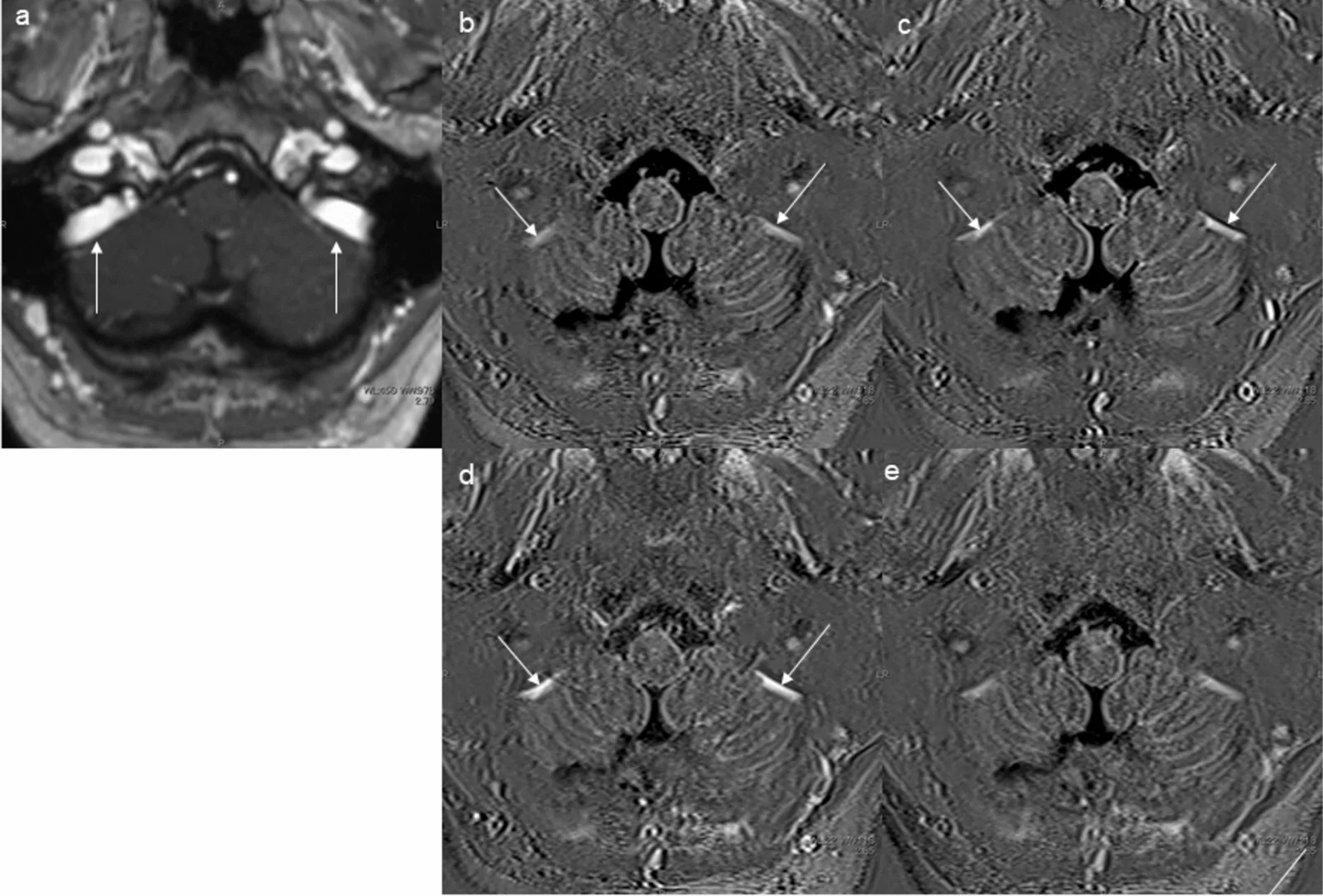
A man in his 60 s who underwent MR examination for the evaluation of endolymphatic hydrops. This is an example of the band-like structures between the sigmoid sinus and cerebellum, which is presumed to be the congested meningeal lymphatic vessel. **a** An MPRAGE image obtained immediately after IV-GBCA. The band-like low signal structures (arrows) are located posterior to the sigmoid sinus. **b**–**e** The time dependent change of the band-like structures after IV-GBCA. The PRISM images obtained pre- administration (**b**), 3 min post-administration (**c**), 4 h after (**d**), and 24 h after (**e**) IV-GBCA. In this subject, the band-like structures with high signal intensity are prominent even before IV-GBCA (arrows, **d**). At 4 h after, the band-like structures show the greatest enhancement. PRISM, Polarity-preserving Real Inversion Solute Mapping; IV-GBCA, intravenous administration of gadolinium-based contrast agent; MPRAGE, magnetization prepared rapid gradient echo

Meningeal lymphatics, perivenous spaces of cortical veins, and pontine perivenous spaces are also depicted as faint white (Fig. 5). These findings suggest that these contents are not pure water [4]. Findings suggestive of meningeal lymphatic stasis around the sigmoid sinus can also be observed without contrast, but become most distinct 4 h after IV-GBCA administration [41] (Fig. 6).

### Evaluation of CSF solute concentration

**Fig. 7:**
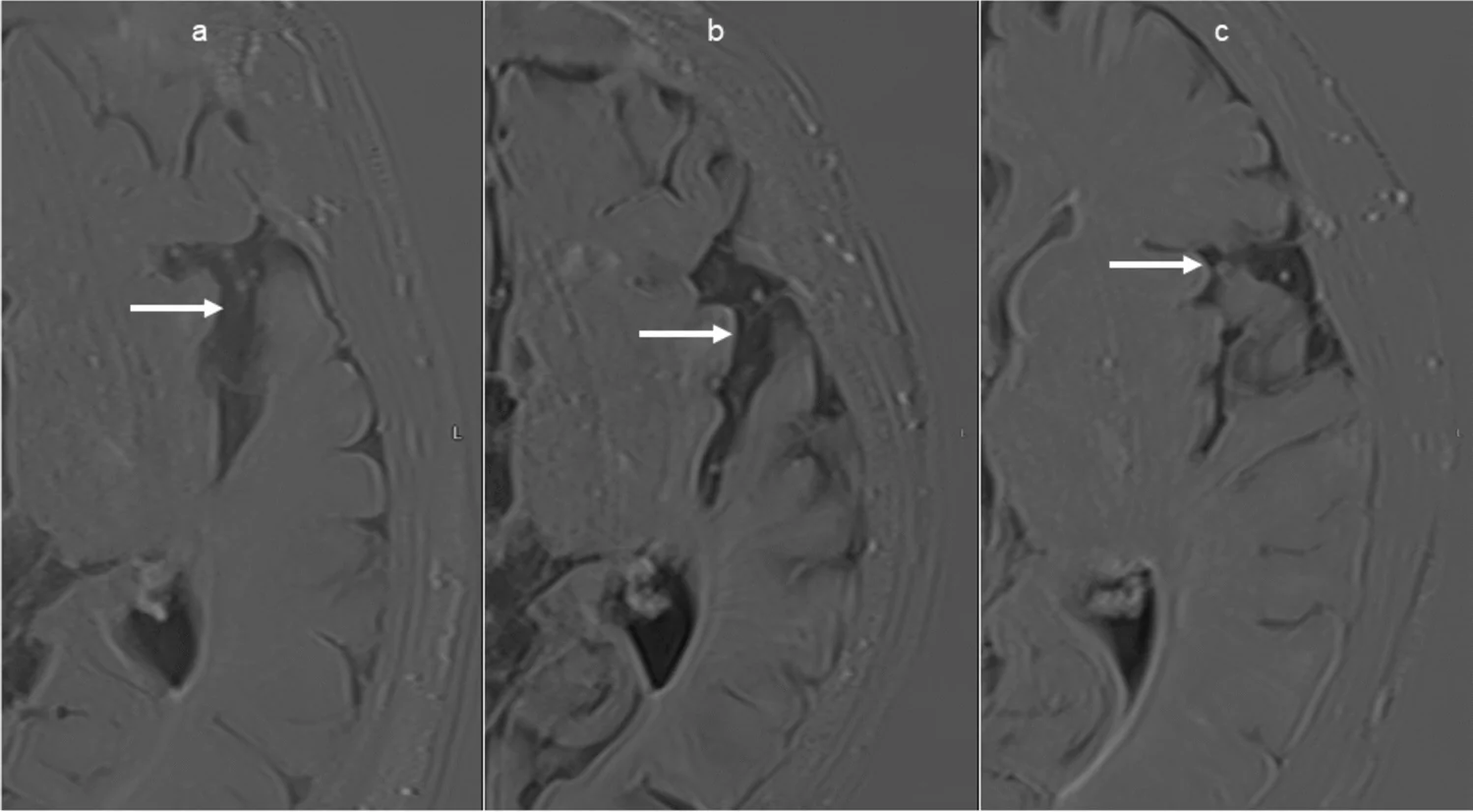
Non-contrast enhanced PRISM images from three patients are shown. The window level and window width are identical for all three images. The left image is from a man in his 70 s (**a**). The signal intensity of cerebrospinal fluid (CSF) within the Sylvian fissure (arrow) is higher than that observed in the other two patients. The middle image is from a woman in her 80 s (**b**). The CSF signal within the Sylvian fissure (arrow) is slightly lower than that of the left case, but higher than that of the right case. The right image is from a woman in her 40 s (**c**). The CSF signal within the Sylvian fissure (arrow) is lower than that of the other two patients. In all cases, the signal intensity of the CSF within the Sylvian fissure is higher than the signal intensity of the CSF within the corresponding lateral ventricles. Subtle differences in signal intensity on non‑contrast PRISM images may reflect variations in cerebrospinal fluid protein concentration. PRISM, Polarity-preserving Real Inversion Solute Mapping

Closer inspection often reveals cases where the CSF signal differs between the subarachnoid space on the brain surface and the ventricles (Fig. 7). This is particularly common in elderly individuals. This is presumed to reflect an increase in protein concentration in the CSF.

### Inner ear assessment

Attempts to evaluate endolymphatic hydrops using non-contrast PRISM [18, 42] have also been reported. While the stable delineation of the endolymphatic space is still difficult with non-contrast imaging, measuring the overall signal of both the endolymphatic and perilymphatic spaces on non-contrast PRISM images has shown a signal decrease in ears with endolymphatic hydrops. This suggests a compositional change in the endolymph in the affected ear.

## Utility of O_2_ inhalation (physiological challenge)

Oxygen has a T1-shortening effect [43]. It is detectable in CSF with conventional FLAIR [6]. In hyperbaric oxygen therapy, signal increase in brain parenchyma, CSF, and vitreous humor has been observed on T1WI and FLAIR in humans. However, the signal increase is particularly prominent on FLAIR [44]. T1 measurement for the purpose of Po_2_ measurement in the vitreous humor is performed with the look-locker sequence [45]. PRISM enables high-sensitivity whole-brain coverage imaging, and it is considered possible to evaluate the distribution to CSF, inner ear lymph fluid, and the eyeball before and after hyperbaric oxygen. Specifically, if the distribution of O_2_ to the inner ear lymph fluid is demonstrated using PRISM, it might approach the mechanism for the effect of Hyperbaric Oxygen Therapy for sudden hearing loss.

## Utility of intravenous GBCA administration

### Time-course observation from early enhancement

By performing time-course imaging with PRISM pre-contrast, immediately post-contrast, 4 h, and 24 h post-contrast, subtle differences in the distribution of GBCA to the CSF in different intracranial regions have been investigated [46]. It has also been shown that the permeability of the blood-perilymph barrier is dependent on age and increases with age [47].

**Fig. 8:**
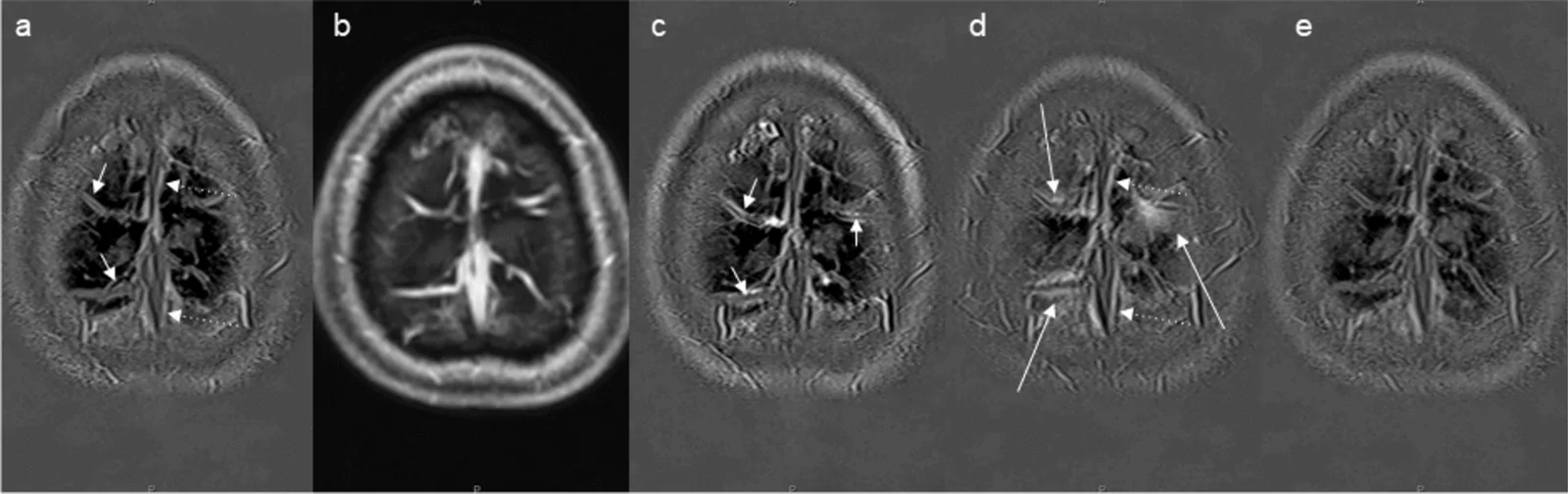
A man in his 60 s with suspected endolymphatic hydrops. On non‑contrast PRISM images (**a**), faint hyperintense sheath‑like structures can be identified surrounding cortical veins and bridging veins (short arrows). Structures presumed to represent meningeal lymphatic vessels along the superior sagittal sinus are also recognizable on non‑contrast PRISM images (dotted arrows). On MPRAGE images obtained immediately after contrast administration (**b**), enhancement of the venous lumen is observed. On PRISM images acquired 10 min after intravenous administration of GBCA (**c**), sheath‑like perivenous enhancement is evident around cortical veins and bridging veins (short arrows). On PRISM images acquired 4 h after contrast administration (**d**), this sheath‑like enhancement is seen to extend into the surrounding subarachnoid space (long arrows). Structures presumed to represent meningeal lymphatic vessels along the superior sagittal sinus show the highest signal intensity and are most conspicuous at this time point (dotted arrows). On PRISM images acquired 24 h after contrast administration (**e**), the enhancement extending into the subarachnoid space appears nearly homogeneous. PRISM, Polarity-preserving Real Inversion Solute Mapping; GBCA, gadolinium-based contrast agent

**Fig. 9:**
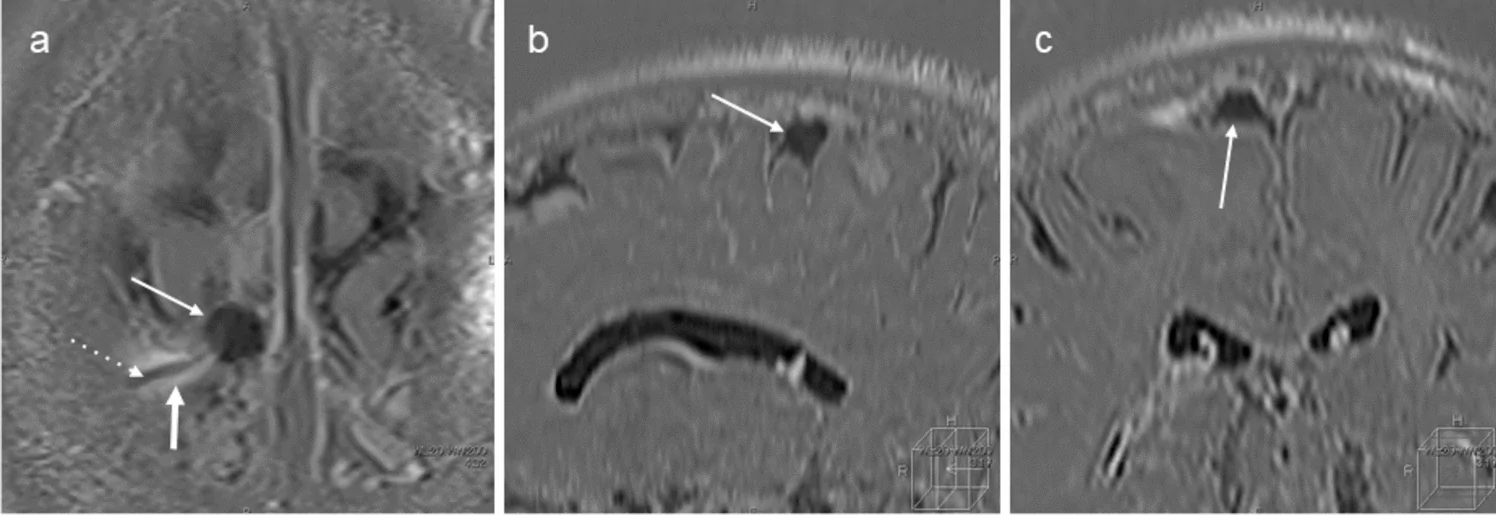
A woman in her 50 s. The axial (**a**), sagittal (**b**) and coronal (**c**) views of PRISM images obtained at 4 h after intravenous administration of GBCA. A cystic area with a long diameter of 10.7 mm (arrows, **a**–**c**) is seen adjacent to the cortical vein (dotted arrow, **a**) and the superior sagittal sinus. GBCA leakage along the cortical vein is visualized (**a**, bold arrow). The cyst is slightly compressing the adjacent brain parenchyma (**a**–**c**). Signals of the cysts are similar to that of cerebrospinal fluid without apparent GBCA distribution. The images are reprinted from the reference under the Creative Commons Attribution-NonCommercial-NoDerivatives International License [53]. PRISM, Polarity-preserving Real Inversion Solute Mapping; GBCA, gadolinium-based contrast agent

**Fig. 10:**
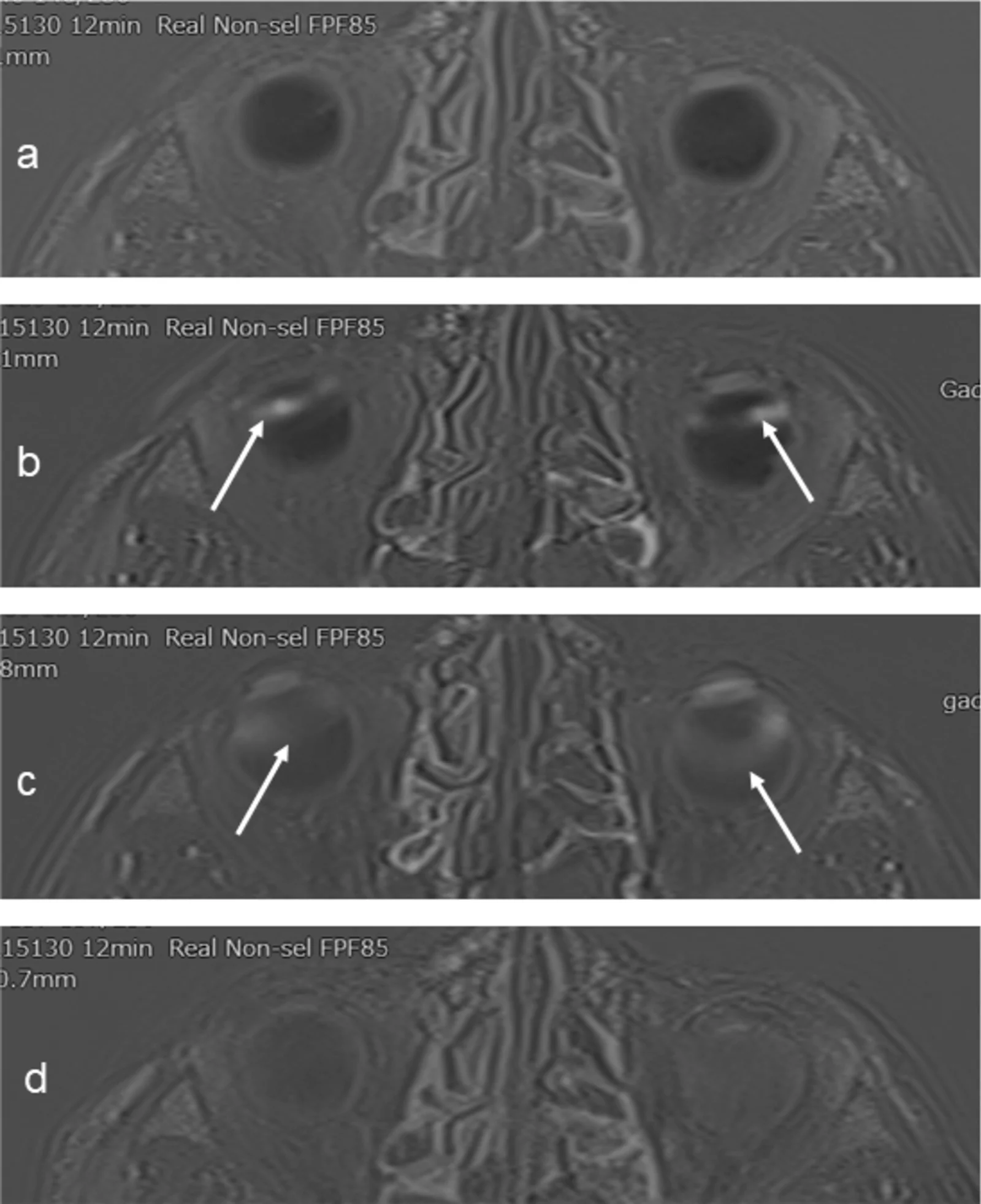
The PRISM images obtained at pre- (**a**) and 10 min (**b**), 4 h (**c**), and 24 h (**d**) after IV-GBCA of a woman in her 70 s with a suspicion of endolymphatic hydrops. These are the axial slices in the lower level of the lens edge. In both eyes, band-like-shaped GBCA leakage into the vitreous from the vicinity of the ora serrata is observed mainly on the temporal side (arrows in **b**) at 10 min after IV-GBCA. The enhancement spread into the vitreous at 4 h after IV-GBCA (arrows in **c**). The band‑like GBCA leakage shows more pronounced posterior migration in the left vitreous body than in the right. This finding suggests lateral asymmetry in the intravitreal tracer dynamics. At 24 h after IV-GBCA, uniform enhancement of the vitreous can be observed **d**). The images are reprinted from the reference under the Creative Commons Attribution-NonCommercial-NoDerivatives International License [27]. PRISM, Polarity-preserving Real Inversion Solute Mapping; GBCA, gadolinium-based contrast agent; IR, inversion recovery; IV, intravenous administration

Furthermore, the utility of PRISM includes the evaluation of blood-labyrinth barrier (BLB) breakdown in various inner ear diseases [48–51], the detection of enhancement in the perivascular spaces of cortical veins on the brain surface in the early phase, and leakage into the surrounding subarachnoid space in the delayed phase [2, 24, 28, 52] (Fig. 8), and the evaluation of the presence of ACES (arachnoid cuff exit sites) cysts and contrast agent retention [20, 53] (Fig. 9). Initial studies evaluating the ocular glymphatic system, in addition to the brain and inner ear, have also reported the discovery of peripheral retinal leakage [27] (Fig. 10). More recently, it has been shown that the direction of movement of this peripheral retinal leakage—whether it moves anteriorly or posteriorly within the vitreous humor over time—is related to the efficiency of contrast agent excretion from the CSF [54]. Meningeal lymphatic stasis posterior to the sigmoid sinus can be observed without contrast, but is most clearly depicted 4 h after IV-GBCA administration [41]. The GBCA washout rate from the CSF is thought to reflect glymphatic function [41]. A relationship has also been reported between the GBCA washout rate and meningeal lymphatic stasis posterior to the sigmoid sinus, as well as the enhancement of perivascular spaces in the basal ganglia [3]. Time-course imaging with PRISM before and after intravenous GBCA administration can provide various information that was previously unknown.

### In the case of delayed enhancement only

Imaging 4 h after intravenous administration is frequently used for the evaluation of endolymphatic hydrops [16, 19, 23, 33, 55, 56]. In cases with short TR conditions of about 6 s, a double dose of intravenous GBCA is used for the stable depiction of endolymphatic hydrops [57].

### Intratympanic GBCA injection

Intratympanic GBCA injection can also be used for evaluating endolymphatic hydrops [21, 25, 58–60]. Although intratympanic GBCA administration is an off-label use, the enhancement effect in the perilymph is often higher with this method than with intravenous gadolinium-based contrast agent administration [61]. Evaluation of endolymphatic hydrops is also becoming possible with intravenous administration, which allows for simultaneous assessment of both ears [62–64]. However, it is generally possible to predict the ear with significant endolymphatic hydrops based on symptoms. Intratympanic GBCA administration also has value as a simulation of the distribution of intratympanically administered drugs. Therefore, the utility of the off-label intratympanic GBCA administration method still exists [62, 64, 65].

## Outlook

Various MRI technical developments are being pursued to evaluate the function of the glymphatic system. DTI-ALPS [66–68] evaluates only a part of the brain’s white matter and is affected by white matter degeneration [69]. More importantly, it is thought to capture the state of water movement only at the time of measurement. The evaluation of the blood-CSF barrier using ASL is also an assessment at the point of measurement [70].

Evaluation using tracers such as ^17^O-labeled water or GBCA requires time-course imaging, both with intrathecal and intravenous administration, which is difficult to perform in many patients [41, 71–75].

A non-contrast method that can evaluate the integrated value of glymphatic system function over a certain long period is necessary for individual diagnosis and screening. Candidates include the evaluation of CSF signal change in each cistern using simple PRISM, evaluation of perivascular spaces in the white matter [4], meningeal lymphatic stasis posterior to the sigmoid sinus [41], and evaluation of the thickness and signal of the meningeal lymphatics near the superior sagittal sinus [76–79].

Previous studies have suggested that non-contrast PRISM contributes to the evaluation of the glymphatic system, reporting findings such as the following. In neurodegenerative diseases such as Alzheimer’s disease, total protein concentrations in the cerebrospinal fluid (CSF) have been shown to increase [80]. Furthermore, in traumatic brain injury, various proteins are transported to the cervical lymphatics via the glymphatic system, including the perivascular space [81]. Because meningeal lymphatic vessels possess physiological characteristics similar to those of peripheral lymph, protein concentrations therein are reported to be substantially higher than those in the interstitial fluid [82, 83].

Integrating these findings with the results from the aforementioned tracer studies and DTI-ALPS would make screening for impaired glymphatic system function and monitoring treatment effects with simple PRISM feasible.

To fully establish the clinical utility of simple PRISM and realize its potential as a key tool for screening and monitoring glymphatic function, the following research priorities must be addressed:

- **Technological standardization:** Establishing short-time, high-resolution PRISM acquisition methods to improve clinical feasibility.
- **Integrative validation:** Simultaneously combining simple PRISM with more time-consuming, yet definitive, quantitative methods (e.g., tracer studies and DTI-ALPS) to systematically validate PRISM’s non-contrast markers.
- **Large-scale evidence collection:** Collecting extensive case data to correlate simple PRISM findings (e.g., CSF signal changes, meningeal lymphatic stasis) with clinical outcomes and measures of impaired glymphatic system function.

A major barrier to the widespread adoption of PRISM is the lack of standardization across different MRI vendors and institutions. Variations in the implementation of “real reconstruction” and differences in 3D-TSE pulse sequence designs (e.g., SPACE, CUBE, VISTA) can affect image contrast. Establishing a unified protocol for TI/TR settings is mandatory for multi-center reproducibility. Furthermore, current PRISM assessments are largely qualitative or semi-quantitative, relying on visual polarity. There is a pressing need to establish quantitative thresholds—for instance, defining specific signal intensity ratios or T1 values that distinguish pathological protein levels from normal variations. Combining PRISM with quantitative techniques like MR Fingerprinting may provide a solution for absolute solute quantification. We sincerely hope this review will encourage researchers globally to adopt simple PRISM and rapidly explore its novel and wide-ranging clinical applications.

## Conclusion

PRISM represents a highly promising approach that enables the evaluation of waste clearance mechanisms in the brain and sensory organs. While this method is poised to advance pathological evaluation to the next stage, its transition into routine clinical practice requires further standardization, broader validation, and confirmed multi-platform reproducibility. Addressing these priorities will be key to establishing PRISM as a reliable diagnostic and monitoring tool in neuroimaging.
